# Computed tomography angiography in the diagnosis of non-ST-elevation myocardial infarction: redefining our first line of defense

**DOI:** 10.1016/j.ijcha.2025.101690

**Published:** 2025-05-02

**Authors:** Yvonne J.M. van Cauteren, Marie-Julie D.K. Lemmens, Sebastiaan C.A.M. Bekkers, Bas L.J.H. Kietselaer, Jordi Heijman, Ralph A.L.J. Theunissen, Braim Rahel, Talitha Voorn, Sander M.J. van Kuijk, Robin Nijveldt, Kevin Vernooy, Joachim E. Wildberger, Casper Mihl, Martijn W. Smulders

**Affiliations:** a Department of Cardiology, Maastricht UMC+, Maastricht, Netherlands (the); b Department of Radiology and Nuclear Medicine, Maastricht UMC+, Maastricht, Netherlands (the); c Cardiovascular Research Institute Maastricht (CARIM), Maastricht University, Maastricht, Netherlands (the); d Department of Cardiology, Bernhoven, Uden, Netherlands (the); e Department of Cardiovascular Medicine, Mayo Clinic, Rochester, MN, United States; f Gottfried Schatz Research Center, Division of Medical Physics & Biophysics, Medical University of Graz, Graz, Austria; g Department of Cardiology, Viecuri Medical Centre, Venlo, Netherlands (the); h Department of Clinical Epidemiology & Medical Technology Assessment (KEMTA), Maastricht UMC+, Maastricht, Netherlands (the); i Department of Cardiology, Radboud University Medical Centre, Nijmegen, Netherlands (the)

**Keywords:** Non-ST-elevation myocardial infarction, Acute chest pain, Diagnostic accuracy, Computed tomography angiography, Vulnerable plaque

## Abstract

**Background:**

Approximately one-third of patients with suspected non-ST-elevation myocardial infarction (NSTEMI) have non-obstructive coronary artery disease. Low-risk patients might benefit from early non-invasive diagnostic testing that can appropriately select those without obstructive coronary artery disease and prevent unnecessary invasive coronary angiography (ICA). The purpose of this study is to evaluate the diagnostic value of computed tomography angiography (CTA) in suspected NSTEMI.

**Methods:**

Patients with clinically suspected type 1 NSTEMI were included. In case ICA was indicated, CTA was performed prior to ICA. The accuracy of CTA to diagnose NSTEMI, assigned by an adjudicated final diagnosis committee, was investigated.

**Results:**

Of the 66 included patients, 40 (61%) were diagnosed with NSTEMI. CAD-RADS ≥ 3 (i.e. stenosis ≥50%) had a sensitivity of 95% (95%CI 83–99%), a specificity of 65% (95%CI 44–83%) and an overall accuracy of 83% (95%CI 72–91%). The Agatston score was significantly different between patients with and without NSTEMI (404 [IQR 132–883] and 31 [IQR 0–163], respectively, p < 0.001). Nineteen patients (29%) met the criteria of ≥2 high-risk plaque (HRP) features, which was more often present in patients with NSTEMI compared to those without NSTEMI (43% and 8%, respectively, p = 0.002). Combining all CTA parameters (CAD-RADS ≥ 3, Agatston score >1.000 and ≥2 HRP features) did not improve the diagnostic accuracy compared with CAD-RADS alone.

**Conclusion:**

CTA accurately diagnoses NSTEMI in patients with acute chest pain and elevated high-sensitivity cardiac troponin T levels. Patients with NSTEMI more often presented with CAD-RADS ≥ 3, Agatston score >1.000 and HRP features.

## 1 Introduction

Diagnosing non-ST-elevation myocardial infarction (NSTEMI) can be difficult. Numerous conditions, including non-coronary related disease, may mimic the clinical presentation of NSTEMI [1]. Up to one third of patients with clinically suspected NSTEMI have myocardial infarction (MI) with non-obstructive coronary arteries (MINOCA) [2,3], which includes dissolved thrombus with or without underlying plaque erosion, type II infarction, coronary spasm, myocarditis or pulmonary embolism. Arguably, patients without obstructive coronary artery disease (CAD) have little benefit from an invasive approach, as it may delay appropriate management or cause procedure-related complications. Routine invasive strategy may only be beneficial for those at the highest risk but is potentially harmful for those at low(er) risk [4]. Therefore, a fast, widely available and accurate non-invasive test to rule out NSTEMI and select appropriate patients for invasive coronary angiography (ICA) could improve current clinical care.

Computed tomography angiography (CTA) is a fast and reliable technique in the work-up of patients with acute chest pain. CTA has a high sensitivity and negative predictive value to rule out CAD in patients with suspected chronic coronary syndromes and patients with low-risk cardiac troponin-negative acute chest pain [[5], [6], [7]]. CTA can also be used to predict prognosis based on calcium score and severity of coronary artery stenosis. The prognostic value can be further increased by investigating high risk plaque (HRP) features and plaque composition [8,9]. Recent studies have evaluated the diagnostic accuracy of CTA in detecting obstructive CAD and its effectiveness in facilitating safe discharge from the emergency department in patients with acute chest pain and elevated troponin levels [3,10]. However, these studies did not investigate the diagnostic accuracy of CTA to detect NSTEMI and therefore carry the risk of underdiagnosing true MINOCA (i.e. MI without a >50% stenosis), potentially leading to under treatment. This study evaluates the diagnostic accuracy of CTA in diagnosing NSTEMI.

## 2 Methods

### 2.1 Study population

**Fig. 1:**
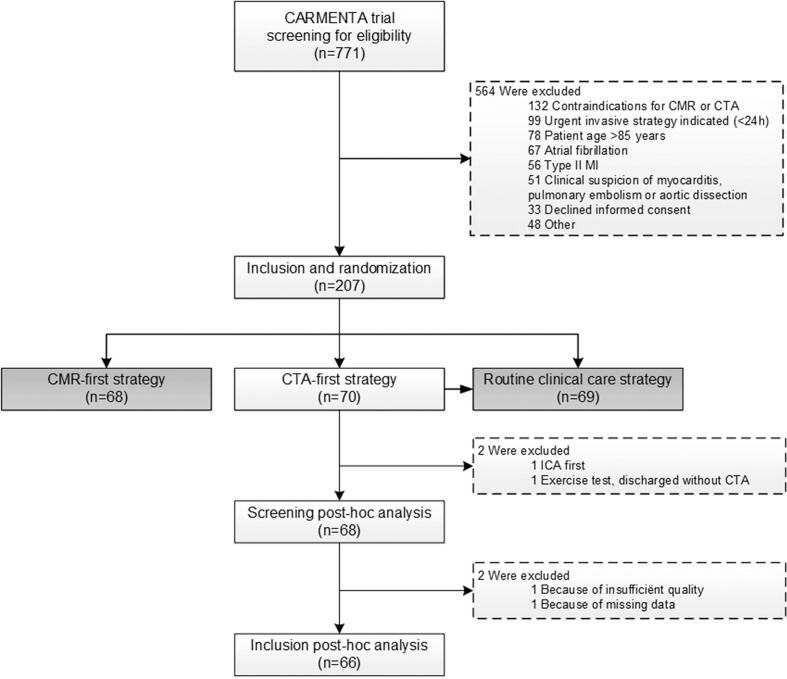
Flow chart. Flowchart of patient inclusion. Abbreviations: CMR = cardiovascular magnetic resonance; CTA = computed tomography angiography; ICA = invasive coronary angiography; MI = myocardial infarct.

This study was a post-hoc analysis of the CARMENTA trial, a randomized controlled clinical study in patients with suspected NSTEMI [2]. The study and imaging protocol have previously been described [11]. Patients referred to the emergency department between April 2012 and May 2016 with acute chest pain, an electrocardiogram (ECG) exam without ST-elevation, and at least one elevated high-sensitivity cardiac troponine T (hs-cTnT) value >14 ng/L were included. Patients with a high suspicion of type II MI (e.g. severe hypertension, atrial fibrillation, heart failure etc.) were excluded [11]. For the current study, patients randomized to the CTA-first strategy were included if they received a CTA as first diagnostic test, followed by (if performed) ICA. One patient was excluded because of insufficient quality and one patient was excluded due to technical issues. Fig. 1 shows the inclusion process.

### 2.2 Objectives

The primary objective of this analysis was to investigate the accuracy of CTA in diagnosing NSTEMI by investigating different CTA parameters. The secondary objective was to investigate differences in plaque composition between culprit (based on ICA results) and non-culprit lesions by using semi-automated plaque quantifications.

### 2.3 Definitions

The final diagnosis of NSTEMI was adjudicated by an independent final diagnosis committee, consisting of an interventional, clinical, and non-invasive imaging cardiologist. All clinical data were reviewed, including results from ICA and available non-invasive diagnostic testing (i.e. ECG, laboratory assessment, cardiac ultrasound, CTA and cardiovascular magnetic resonance imaging). NSTEMI was diagnosed according to the universal definition of MI [12]. Alternative diagnoses were non-coronary (i.e. definitely no coronary etiology but no clear other diagnosis), non-coronary but cardiac (e.g. myocarditis, valvular disease), non-cardiac, or unknown etiology.

### 2.4 CTA scan protocol

CTA was performed using a second generation dual-source CT-scanner (Somatom Definition Flash; Siemens Healthineers, Forchheim, Germany) <72 hours after admission. A non-contrast CT scan was acquired to determine coronary calcium score [10]. A contrast-enhanced triple rule-out angiography protocol using an iodinated contrast agent (Ultravist 300; Bayer Pharma AG, Berlin, Germany) was performed to visualize coronary arteries, pulmonary arteries, and the aorta within a single acquisition. A prospective high-pitch spiral protocol was used in patients with a stable heart rate <65 bpm, whilst a retrospective dose modulation helical protocol was performed in all other patients.

### 2.5 CTA analysis

**Fig. 2:**
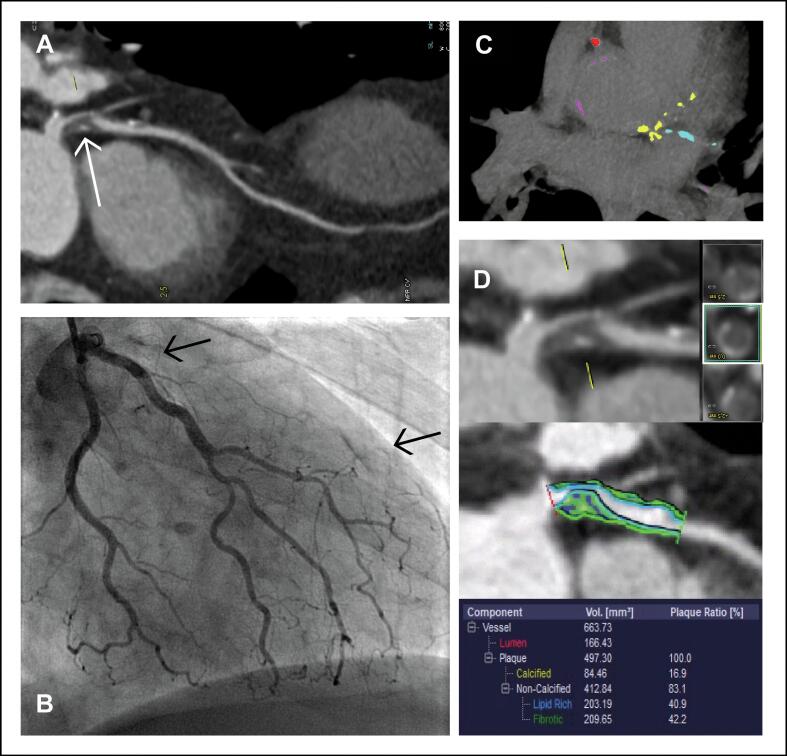
Patient example. Example of a patient presenting with non-ST-elevation myocardial infarction. A computed tomography angiography (panel A, C and D) was performed prior to invasive coronary angiography (panel B). Panel A: coronary artery stenosis (CAD-RADS 4A) of the proximal left anterior descending artery (**white arrow**). Panel B: proximal total occlusion of the left ascending descending artery with collateral filling (**black arrows**). Panel C: high Agatston score. Panel D: high-risk plaque with napkin-ring sign and low attenuation, and higher ratios of fibrosis and lipid compared to calcified (quantitative plaque analyses).

*Visual assessment.* A dedicated post-processing workstation (Syngo.via, Siemens Healthineers) was used. Images were reviewed in consensus by two experienced observers, one cardiovascular radiologist (CM, 14 years of experience) and one imaging cardiologist (MS, 13 years of experience), following SCCT guidelines [13]. The observers were blinded for clinical and ICA data. CTA scans were analysed using the Coronary Artery Disease-Reporting and Data System (CAD-RADS) 2.0 score [14]. The degree of stenosis was determined for each segment. A CAD-RADS score of ≥3 was considered as obstructive CAD. HRP features were scored in all patients and included napkin-ring sign, outward remodelling, spotty calcifications, and low attenuation of the plaque. A plaque was considered high-risk when ≥2 HRP features were present [15]. Fig. 2 presents a typical example of a patient**.**

*Semi-automated plaque quantification*. Semi-automated plaque quantification software (Coronary Plaque Analysis 4.2.0, Syngo.via, Siemens Healthineers) was used to assess plaque composition. In patients with NSTEMI, all plaques with CAD-RADS ≥ 3 and a vessel diameter ≥2 mm were analysed. The software automatically delineates the vessel wall, lumen, and plaque, followed by manual adjustment if necessary. Hounsfield unit values between −100 and 69 were considered as lipid, values between 70 and 149 as fibrotic, and values between 150 and 950 as calcified components [16]. Lipid, fibrotic, and calcified volumes were extracted to calculate ratios.

### 2.6 ICA analysis

In a separate analysis, stenosis severity as visualized with ICA was assessed by an interventional cardiologist (RT, 12 years experience), who was blinded for the CTA results. The degree of segment stenosis was scored similar to the CTA analysis (0%, 1–24%, 25–49%, 50–69%, 70–99% and 100%). The culprit lesion was scored for all patients with NSTEMI and was assigned during the index event based on ICA results in combination with available non-invasive imaging.

### 2.7 Statistical analysis

Statistical analyses were performed using SPSS statistics (25.0; IBM, Chicago, Illinois). Categorical values are presented as numbers and frequencies and compared using Pearson’s chi-squared or Fisher’s exact test. Continuous variables are given as mean ± standard deviation when normally distributed, or median with interquartile range when not normally distributed, and were compared using the independent-samples *T*-test or the Mann-Whitney *U* test, respectively. Statistical significance was assumed with a p-value ≤ 0.05.

The accuracy of CTA in diagnosing NSTEMI was assessed by calculating sensitivity, specificity, positive predictive value, negative predictive value, and overall accuracy including their 95% confidence interval (CI), using the final diagnosis committee as reference. These analyses were performed for the following CTA parameters: CAD-RADS ≥ 3, CAD-RADS ≥ 4, HRP features, and Agatston score >1.000. Additionally, these parameters were combined to investigate if the diagnostic accuracy could be improved. All possible combinations were tested and were considered positive when at least one CTA parameter was abnormal.

In a subset of patients with NSTEMI and CAD-RADS ≥ 3, semi-quantitative plaque analysis was applied. For this analysis, patients should have at least two stenoses ≥50%. Plaque composition (based on lipid, fibrotic and calcified ratios) was used to differentiate between culprit and non-culprit lesions (as identified on ICA). The paired-samples T-test was used to assess the difference in ratios.

## 3 Results

### 3.1 Baseline characteristics

A total of 66 patients with suspected NSTEMI were included (see Fig. 1). Detailed baseline characteristics are shown in Table 1. In short, 73% of patients were male and the average age was 65 years. ICA was performed in 45 patients (68%), of whom 36 showed a stenosis ≥70% and were revascularized.

**Table 1:** Baseline characteristics.

	Total sample (n = 66)	Without NSTEMI* (n = 26)	NSTEMI (n = 40)	p-value
**General**				
Age (years)	64.6 ± 12.9	62.3 ± 16.7	66.0 ± 9.7	0.307
Male	48 (73)	14 (54)	34 (85)	**0.005**
BMI (kg/m^2^)	26.4 ± 4.0	26.7 ± 4.0	26.2 ± 4.0	0.622
**Risk factors**				
Hypertension	29 (44)	11 (42)	18 (45)	0.830
Hypercholesterolemia	16 (24)	9 (35)	7 (18)	0.113
Family history	26 (39)	8 (31)	18 (45)	0.248
Smoking	22 (33)	6 (23)	16 (40)	0.154
Diabetes mellitus	4 (6)	3 (12)	1 (3)	0.292
Total number of risk factors	1.5 ± 1.2	1.4 ± 1.2	1.5 ± 1.1	0.860
**CV disease**				
CVA/TIA	7 (11)	4 (15)	3 (8)	0.420
PVD	4 (6)	1 (4)	3 (8)	1.000
**GRACE risk score**				0.833
Low risk (≤108)	28 (42)	10 (39)	18 (45)	
Intermediate risk (109–140)	30 (46)	13 (50)	17 (43)	
High risk (>140)	8 (12)	3 (12)	5 (13)	
GRACE score	116 ± 22	114 ± 23	117 ± 22	0.600
**Laboratory assessment**				
Hs-cTnT baseline (ng/L)	50 (25–92)	31 (24–59)	61 (27–120)	0.075
Hs-cTnT after 3 h (ng/L)	59 (25–194)	35 (22–67)	105 (36–231)	**0.003**
**Electrocardiogram**				
Normal or inconclusive	46 (70)	20 (77)	26 (65)	0.303
**ICA results**				
No ICA performed	21 (32)	19 (73)	2 (5)	NA
Non-significant CAD	9 (15)	7 (27)#	2 (5)	NA
1-vessel disease	15 (23)	0 (17)	15 (34)	NA
2-vessel disease	8 (18)	0 (0)	8 (21)	NA
3-vessel disease/left main	13 (20)	0 (0)	13 (33)	NA
CAD ≥ 70% stenosis	36 (55)	0 (0)	36 (90)	**<0.001**
Revascularisation during index event	36 (55)	0 (0)	36 (90)	**<0.001**
**Culprit based on ICA**				
Left main artery	NA	NA	1 (3)	NA
Left anterior descending artery	NA	NA	14 (35)	NA
Circumflex artery	NA	NA	8 (20)	NA
Right coronary artery	NA	NA	11 (28)	NA
Unknown	NA	NA	4 (10)	NA

Categorical data presented as frequencies (percentages), continuous data presented as mean ± standard deviation or median (range).

Abbreviations: BMI = body mass index; CAD = coronary artery disease; CV = cardiovascular; CVA = cerebrovascular accident; hs-cTnT = high-sensitivity cardiac troponin T; ICA = invasive coronary angiography; NA = not applicable; NSTEMI = non-ST-elevation myocardial infarction; PVD = peripheral vascular disease; TIA = transient ischemic attack.

* *Alternative diagnoses:* chest pain of non-coronary etiology (n = 8), non-cardiac etiology (n = 5), pulmonary embolism (n = 2), non-coronary but cardiac etiology including (peri-)myocarditis based on an additional cardiac magnetic resonance imaging (n = 5), valvular aortic stenosis (n = 1), left ventricular hypertrophy (n = 1), and the remainder as unknown diagnosis (n = 3).

# Two of these patients had CAD-RADS 3 on CTA but were still referred for ICA by the threating cardiologist, and 5 patients had CAD-CADS ≥ 4 but non-significant CAD on ICA.

According to the final diagnosis committee, NSTEMI was present in 40 patients (61%), of whom 36 (90%) had ≥70% stenosis on ICA. The diagnoses of the remaining 26 patients (39%) without NSTEMI are described in Table 1. Baseline characteristics between patients with and without NSTEMI were relatively similar except for a higher proportion of males (85% vs 54%, p = 0.005, respectively) and higher hs-cTnT levels after 3 hours in NSTEMI patients.

### 3.2 CTA results

In the total study population, CAD-RADS score ≥3 was observed in 47 patients (71%), of whom 4 patients (6%) had CAD-RADS 3 and 43 (65%) CAD-RADS ≥ 4. Nineteen patients (29%) had CAD-RADS < 3. Out of 40 patients diagnosed with NSTEMI, one patient (3%) had CAD-RADS 3, 37 patients (93%) had CAD-RADS ≥ 4, and two patients (5%) had CAD-RADS < 3 on CTA. In patients without NSTEMI (n = 26), three patients (12%) had CAD-RADS 3, six patients (23%) CAD-RADS ≥ 4, and 17 patients (65%) had CAD-RADS < 3 (see Table 2).

**Table 2:** CTA characteristics.

	Total population (n = 66)	Without NSTEMI (n = 26)	NSTEMI (n = 40)	p-value
**CAD-RADS ≥ 3 (≥50%)**	47 (71)	9 (35)	38 (95)	**<0.001**
**CAD-RADS ≥ 4 (≥70%)**	43 (65)	6 (23)	37 (93)	**<0.001**
**Agatston score**				
Median (IQR)	212 (8–570)	31 (0–163)	404 (132–883)	**<0.001**
Score of 0	13 (20)	9 (35)	4 (10)	**0.014**
Score of >1.000	8 (13)	0 (0)	8 (20)	**0.015**
**High-risk plaque features**				
Outward remodelling	24 (36)	3 (12)	21 (53)	**<0.001**
Spotty calcification	19 (29)	4 (15)	15 (38)	0.080
Low attenuation	11 (17)	1 (4)	10 (25)	**0.015**
Napkin ring sign	8 (12)	1 (4)	7 (18)	0.097
Plaque with ≥2 features	19 (29)	2 (8)	17 (43)	**0.002**
**Other significant findings**				
Pulmonary embolism	2 (3)	2 (8)	0 (0)	0.149

Categorical data presented as frequencies (percentages), continuous data as median (IQR).

Abbreviations: CAD-RADS = coronary artery disease-reporting and data system; IQR = interquartile range; NSTEMI = non-ST-elevation myocardial infarction

The median Agatston score was 212 (IQR 8–570). The Agatston score was significantly higher in patients with NSTEMI than without NSTEMI (404 IQR [132–883] and 31 IQR [0–163], respectively, p < 0.001). Eight patients in the NSTEMI group had an Agatston score >1.000, compared to none of the patients without NSTEMI (p = 0.015). Thirty-three patients (50%) of this cohort had at least one HRP feature. A napkin-ring sign was observed in eight patients (12%), outward remodelling in 24 patients (36%), spotty calcifications in 19 patients (29%), and low attenuation plaque in 11 patients (17%). Nineteen patients (29%) met the criteria of ≥2 HRP features within one plaque. This was more often seen in patients with NSTEMI compared to those without NSTEMI (43% and 8%, respectively, p = 0.002) (see Table 2).

### 3.3 Accuracy of CTA to diagnose NSTEMI

**Fig. 3:**
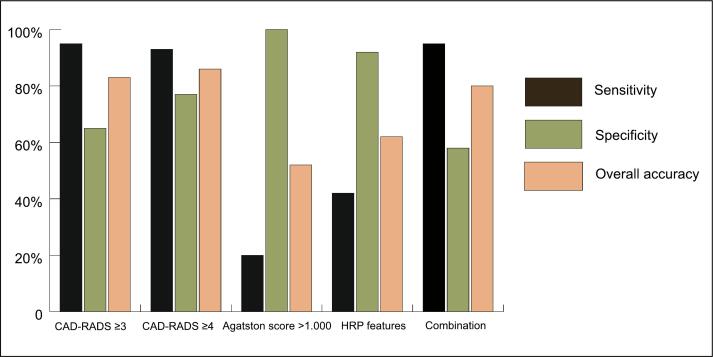
Diagnostic accuracy. Accuracy of computed tomography angiography to diagnose non-ST-elevation myocardial infarction, based on CAD-RADS score, Agatston score >1.000 and high-risk plaque features. Abbreviations: CAD-RADS = coronary artery disease-reporting and data system; CTA = computed tomography angiography; HRP = high-risk plaque.

Table 3 visualizes the diagnostic accuracy results of CTA in diagnosing NSTEMI. The presence of CAD-RADS ≥ 3 had a sensitivity of 95% (95%CI 83–99%), a specificity of 65% (95%CI 44–83%) and an overall accuracy of 83% (95%CI 72–91%) when diagnosing NSTEMI. CAD-RADS ≥ 4 had a sensitivity of 93% (95%CI 80–98%), specificity of 77% (95%CI 56–91%) and overall accuracy of 86% (95%CI 75–93%). An Agatston score >1.000 had a sensitivity of 20% (95%CI 9–36%), specificity of 100% (95%CI 87–100%), and overall accuracy of 52% (95%CI 39–64%) to diagnose NSTEMI. The presence of ≥2 HRP features had a sensitivity of 43% (95%CI 26–58%), specificity of 92% (95%CI 75–99%), and overall accuracy of 62% (95%CI 49–74%). The combination of CAD-RADS ≥ 3, Agatston score >1.000, and HRP features (i.e. at least one positive) had a sensitivity of 95% (95% CI 83–99%), specificity of 58% (95%CI 37–77%) and overall accuracy of 80% (95%CI 69–89%) to diagnose NSTEMI (see Fig. 3). For the other combinations see Table 4.

**Table 3:** Diagnostic accuracy of CTA to diagnose NSTEMI.

	Sensitivity(%) (95% CI)	Specificity(%) (95% CI)	PPV(%) (95% CI)	NPV(%) (95% CI)	Overall accuracy (%) (95% CI)
CAD-RADS ≥ 3 (≥50%)	95 (83–99)	65 (44–83)	81 (71–88)	89 (68–97)	83 (72–91)
CAD-RADS ≥ 4 (≥70%)	93 (80–98)	77 (56–91)	86 (75–93)	87 (69–95)	86 (75–93)
Agatston score >1.000	20 (9–36)	100 (87–100)	100 (63–100)	44 (41–49)	52 (39–64)
HRP features	43 (26–58)	92 (75–99)	89 (68–97)	51 (44–58)	62 (49–74)
Combination CAD-RADS ≥ 3, Agatston score >1.000 and ≥2 HRP features*	95 (83–99)	58 (37–77)	78 (69–84)	88 (65–97)	80 (69–89)

Abbreviations: CAD = coronary artery disease; CAD-RADS = coronary artery disease-reporting and data system; CI = confidence interval; CTA = computed tomography angiography; HRP = high-risk plaque; NPV = negative predictive value; NSTEMI = non-ST-elevation myocardial infarction; PPV = positive predictive value.

* Scored positive when at least one parameter positive.

**Table 4:** Diagnostic accuracy of different combinations of CTA parameters to diagnose NSTEMI.

CTA parameters*	Sensitivity(%) (95% CI)	Specificity (%) (95% CI)	PPV(%) (95% CI)	NPV(%) (95% CI)	Overall accuracy (%) (95% CI)
CAD-RADS ≥ 3 or ≥2 HRP features	95 (83–99)	58 (37–77)	78 (69–84)	88 (65–97)	80 (69–89)
CAD-RADS ≥ 3 or Agatston >1.000	95 (83–99)	65 (44–83)	81 (71–88)	89 (68–97)	83 (71–91)
CAD-RADS ≥ 4 or ≥2 HRP features	93 (80–98)	69 (48–86)	82 (72–89)	86 (66–95)	83 (71–91)
CD-RADS ≥ 4 or Agatston >1.000	93 (80–98)	77 (56–91)	86 (75–93)	87 (69–95)	86 (76–94)
CAD-RADS ≥ 4, Agatston score > 1.000 or ≥2 HRP features	92 (79–98)	69 (48–86)	82 (72–89)	86 (66–95)	83 (72–91)

Abbreviations: CAD-RADS = coronary artery disease-reporting and data system; CI = confidence interval; CTA = coronary computed tomography angiography; HRP = high-risk plaque features; NPV = negative predictive value; NSTEMI = non-ST-elevation myocardial infarction; PPV = positive predictive value.

* Scored positive when at least one parameter positive.

### 3.4 CTA plaque characterization of the culprit coronary artery

**Fig. 4:**
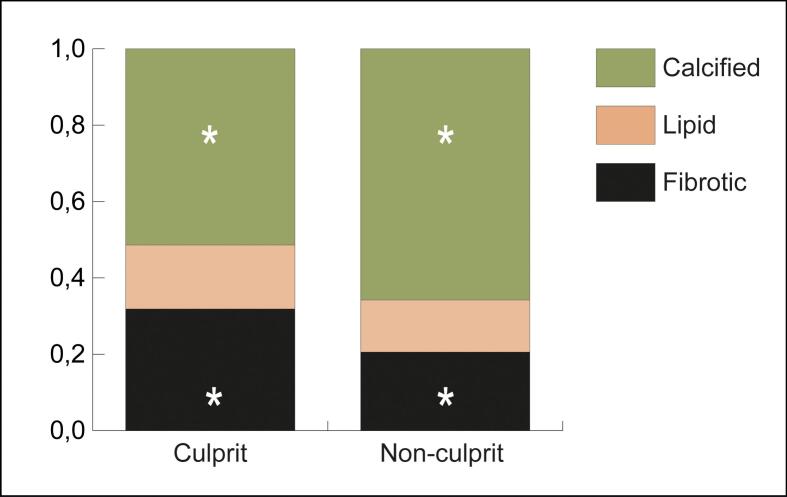
Plaque composition. Comparison of plaque composition assessed on computed tomography angiography of culprit and non-culprit plaques. * (**asterisk**): significantly different between culprit and non-culprit.

Twenty-two patients had more than one lesion and were included in the analysis. Culprit lesions identified on ICA had significantly higher fibrotic and lower calcified ratios on CTA compared with non-culprit plaques, as described in Table 5 and presented in Fig. 4.

**Table 5:** Composition culprit versus non-culprit (ratios).

	Culprit	Non-culprit	Mean difference	P-value	95% CI interval	
					Lower	Upper
Calcified ratio	0.5139	0.6580	−0.14413	**0.019**	−0.26151	−0.02675
Lipid ratio	0.1675	0.1364	0.03108	0.223	−0.02040	0.08256
Fibrotic ratio	0.3186	0.2056	0.11303	**0.005**	0.03836	0.18769

Abbreviations: CI = confidence interval.

## 4 Discussion

This study demonstrates that CTA can accurately diagnose NSTEMI, achieving a sensitivity of 95%. The specificity for CAD-RADS ≥ 3 was 65%. Although patients with NSTEMI were significantly more likely to have Agatston scores >1.000 and HRP features, incorporating these parameters (individually or combined) into the analysis did not improve diagnostic accuracy. Culprit plaques exhibited higher fibrotic and lower calcified ratios compared to the non-culprit plaques.

CTA is a well-established diagnostic tool in patients with stable angina pectoris because of its high negative predictive value [17,18]. The ROMICAT-II trial investigated whether CTA could be incorporated into the emergency department for low-risk acute chest pain patients without troponin elevation or ECG changes suggestive of NSTEMI [6]. More recently, the VERDICT trial showed a high diagnostic performance to rule out (negative predictive value 90.9%) and rule in (positive predictive value 87.9%) significant CAD (coronary artery stenosis ≥50%) in NSTEMI patients [3]. Both the ROMICAT-II and VERDICT trial used obstructive CAD as an endpoint, while the current study investigated NSTEMI as primary endpoint. A study by Arslan et al. investigated the diagnostic value of CTA in a population with only moderately elevated high-sensitivity cardiac troponin levels (i.e. ‘observe zone’) for diagnosing acute coronary syndrome. They reported a sensitivity of 95% (95%CI 74–100) and specificity of 56% (95%CI 45–68) which is comparable to our findings.

It is worth noting that in the study by Arslan et al. of the 106 included patients, only 20 patients (19%) had an adjudicated diagnosis of type I non-ST-segment elevation acute coronary syndrome [19]. Numerous conditions, including non-coronary related disease, may mimic the clinical presentation of NSTEMI. Diagnosing NSTEMI solely based on history taking, risk factors, ECG and mildly elevated high-sensitivity cardiac troponin levels is therefore challenging. This is also reflected by a study published by Shanmuganathan et al [20] in patients with higher risk suspected NSTEMI, performing Ccardiovascular magnetic resonance imaging prior to ICA. Only 67% of patients had a definite diagnosis of MI, including 6 (6%) with true MINOCA (confirming our results: 61% and 6%, respectively). In addition to prior research, our study supports the role of CTA as first diagnostic test in patients with suspected NSTEMI. Although the systematic use of CTA in patients with suspected NSTEMI is implied, more studies are warranted whether this strategy could be performed in all patients or only in certain subgroups of patients.

The prevalence of ≥2 HRP features was nearly 30%, aligning with the rates reported in the expert consensus document [14]. HRP features have been associated with a higher risk of future acute coronary syndrome [21,22]. More recently Suzuki et al. showed a strong correlation between HRP features on CTA and thin-cap fibroatheroma detected by optical coherence tomography [23]. Thin-cap fibroatheroma is an independent predictor of future cardiac events [24]. The studies of Motoyama et al. [21] and Puchner et al. [22] were performed in patients with either stable chest pain or chest pain without elevated troponin levels. CTA was performed prior to ICA in the current study, ensuring that the presence of HRP features and stenosis severity was not influenced by any prior interventions. It remains unclear how NSTEMI and its medical treatment affect HRP features. These features may be altered by plaque rupture or by the medication these patients received. Additionally, stenting can potentially ‘seal’ the plaque, contributing to its stabilization.

Semi-quantitative software was used to investigate whether plaque composition differed between culprit and non-culprit lesions. Culprit plaques showed higher fibrotic and lower calcified ratios when compared to non-culprit plaques. These results are comparable with the study of Tesche et al. [25], and in line with literature stating that at-risk plaques have higher non-calcified volumes and lower calcified volumes [26,27] since these plaques are more prone to rupture, thereby causing MI. The strength of the current study is the comparison of culprit and non-culprit plaques in the same patients, to avoid bias introduced by inter-patient differences, despite this reduced the sample size as it could be only performed in patients with ≥2 lesions. Although the results in our study are promising, they must be interpreted with caution since the result cannot yet be extrapolated to a larger group of patients. Furthermore, the used software is not yet validated and time consuming. This could potentially be overcome in the future by using artificial intelligence [28].

A triple rule-out scan was performed, highlighting one of the key advantages of non-invasive imaging over an invasive strategy: the ability to identify a broader range of (alternative) diagnoses, such as a pulmonary embolism or aortic dissection [29]. Indeed, two patients were diagnosed with pulmonary embolism based on CTA findings. This number is lower than comparable studies. One potential explanation for this finding is that patients with a high suspicion of type II MI were excluded. Despite these advantages, a triple rule out scan increases radiation exposure and contrast media volumes. In addition, the diagnostic accuracy for obstructive CAD is slightly lower compared to a dedicated coronary CTA. The slightly higher yield for pulmonary embolism or aortic dissection does not outweigh the limitations of a triple-out scan, indiscriminate use is not warranted [30].

A promising novel imaging technique is photon-counting detector CT. By counting individual incoming photons and measuring their energy, it allows for better X-ray photon energy separation, thereby improving differentiation of tissue types and materials. The potential benefits are enhanced plaque characterisation, improved visualisation of small calcifications and improved in-stent lumen assessment [31]. The diagnostic accuracy of CTA could also be improved by using CTA-derived fractional flow reserve (FFR), enabling non-invasive assessment of the hemodynamic significance of CAD [32]. The NXT trial showed that CTA-derived FFR measurements provide a high diagnostic accuracy for hemodynamically significant CAD with invasive FFR as the reference standard [33]. Patients with stable angina pectoris and a normal CTA-derived FFR test (i.e. ≤0.80) have a low 3-years risk of all-cause death and nonfatal MI [34]. Incorporating CTA-derived FFR measurements into the standard CTA protocol – particularly when using photon-counting detector CT – has significant potential to further improve the diagnostic accuracy of CTA for detecting NSTEMI.

In summary, a CAD-RADS score of ≥3 on CTA can accurately detect NSTEMI in patients presenting with acute chest pain, elevated hs-cTnT levels, and a normal or inconclusive ECG. While patients with NSTEMI more often had an Agatston score >1.000 and HRP features, incorporating these features in the analysis did not improve diagnostic accuracy. Culprit plaques demonstrated a higher fibrotic and lower calcified ratio when compared to non-culprit plaques. CTA shows great promise as first-line diagnostic tool in patient with suspected NSTEMI and could serve as a useful gatekeeper for ICA.

## CRediT authorship contribution statement

**Yvonne J.M. van Cauteren:** Writing – review & editing, Writing – original draft, Software, Methodology, Formal analysis. **Marie-Julie D.K. Lemmens:** Writing – review & editing, Methodology. **Sebastiaan C.A.M. Bekkers:** Writing – review & editing, Conceptualization. **Bas L.J.H. Kietselaer:** Writing – review & editing, Conceptualization. **Jordi Heijman:** Writing – review & editing, Methodology, Conceptualization. **Ralph A.L.J. Theunissen:** Writing – review & editing, Software, Formal analysis. **Braim Rahel:** Writing – review & editing. **Talitha Voorn:** Writing – review & editing, Visualization, Formal analysis. **Sander M.J. van Kuijk:** Writing – review & editing, Methodology, Formal analysis. **Robin Nijveldt:** Writing – review & editing, Funding acquisition. **Kevin Vernooy:** Writing – review & editing. **Joachim E. Wildberger:** Writing – review & editing, Conceptualization. **Casper Mihl:** Writing – review & editing, Writing – original draft, Supervision, Software, Methodology, Formal analysis, Conceptualization. **Martijn W. Smulders:** Writing – review & editing, Writing – original draft, Supervision, Methodology, Funding acquisition, Formal analysis, Conceptualization.

## Funding

This manuscript contains post-hoc analysis of the CARMENTA trial. This study has received funding from Netherlands Heart Foundation (grant 2014T051). In addition, this work was supported by an Academic Alliance Fund 2020 (Maastricht UMC+ and Radboud UMC).

## Declaration of competing interest

The authors declare the following financial interests/personal relationships which could be considered as potential competing interests: Martijn W. Smulders reports financial support for the CARMENTA trial provided by Netherlands Heart Foundation. Martijn W. Smulders and Casper Mihl received funding (2024) of a collaborative project co-financed with PPP allowance made available by Health-Holland, Top sector Life Sciences & Health to stimulate public-private partnerships and funding from Siemens Healthineers Nederlands B.V.. Joachim E. Wildberger reports a relationship with Siemens Healthineers AG that includes consulting or advisory and speaking and lecture fees. Jordi Heijman is Associate editior of International Journal of Cardiology Heart & Vasculature. Casper Mihl is part of the editorial board of International Journal of Cardiology Heart & Vasculature. If there are other authors, they declare that they have no known competing financial interests or personal relationships that could have appeared to influence the work reported in this paper.
